# cpalockator: Thread-Modular Analysis with Projections (Competition Contribution)

**DOI:** 10.1007/978-3-030-72013-1_25

**Published:** 2021-02-26

**Authors:** Pavel Andrianov, Vadim Mutilin, Alexey Khoroshilov

**Affiliations:** 8 Eindhoven University of Technology, Eindhoven, The Netherlands; 9 Aalborg University, Aalborg East, Denmark; 10 Ivannikov Institute for System Programming of RAS, Moscow, Russia; 11 Lomonosov Moscow State University, Moscow, Russia; 12 Moscow Institute of Physics and Technology, Moscow, Russia; 13 Higher School of Economics, Moscow, Russia

**Keywords:** Multithreading, Projection, Thread-modular approach

## Abstract

Our submission to SV-COMP’21 is based on the software verification framework  and implements the extension to the thread-modular approach. It considers every thread separately, but in a special environment which models thread interactions. The environment is expressed by projections of normal transitions in each thread. A projection contains a description of possible effects over shared data and synchronization primitives, as well as conditions of its application. Adjusting the precision of the projections, one can find a balance between the speed and the precision of the whole analysis.

Implementation on the top of the  framework allows combining our approach with existing algorithms and analyses. Evaluation on the sv-benchmarks confirms the scalability and soundness of the approach.

## 1 Verification Approach

The main challenge for verification of industrial multithreaded software is to consider a potential thread interaction efficiently. Our verification approach is based on the thread-modular technique [4,5]. The approach allows avoiding a cartesian product of thread states by considering each thread state separately. Thus, an abstract state is not a complete one anymore and represents only one thread in a partial abstract state. However, due to this, the analysis has no information about transitions in other threads, which are strongly required for the soundness of the analysis. Thus, to not lose soundness we have to take into account the influence of other threads to the considered thread. For that purpose, we compute a special representation of the environment, which consists of a set of thread transitions, so-called projected transitions, or *projections*. The projections may be more or less precise, which strongly affects the precision and speed of the whole analysis. Note, the projections are independent and thus, a correct

⋆ Representing jury member, corresponding author: andrianov@ispras.ru sequence is missed. Potentially, all projections may affect the other thread in any time. It is an overapproximation, leading to an imprecise analysis.

Let us explain, how we increase precision considering only *compatible* projections.

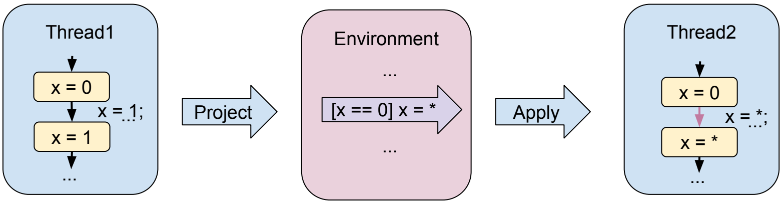

**Fig. 1.** Computation of a thread environment and its application

The figure 1 shows one step of the analysis. After computation of an abstract state in the first thread, we should spread the effect ($x$ is a shared variable) to the other threads. Thus, we compute a *projection* of the operation. The projection is a part of the environment and affects the other threads through it. Then we *apply* a new effect to the other threads.

In the example, we lose the precision of the effect, abstracting from the assigned value ($x = *$). One of the key ideas of the proposed approach is to extend abstraction not only to states but also to operations, i.e. transitions. Thus, the projection may look like $x = 1$ and $* = *$ in other configurations. That allows adjusting the level of abstraction of the environment for a specific task. By adjusting the configuration it is possible to vary not only an abstraction level but also to construct an algorithm that may be closer either to data-flow analysis or to software model checking.

To be able to construct precise analysis we suggest to encode not only abstract operations but also some conditions of its application, so-called *guards*. The guards are related to a predecessor abstract state, but they are not required to be equal to it. The guards store some information about variable values, locks, threads, or even abstract predicates. In the figure 1 the guard contains information about the initial value of the modified variable $x$ ($x == 0$). A projection may be applied to a particular state if the guards allow it. We say, that the projection is *compatible* to an abstract state of the other thread. In our example the effect $x = *$ may be applied to the other thread only if the corresponding state does not contradict the condition $x == 0$.

More information about the approach and theoretical preliminaries can be found in [1]. Practical application of the theory to the Linux kernel drivers can be found in [2].

## 2 Software Architecture

CPALockator is based on the CPAchecker framework and has the same software architecture. Its key concept is CPA [3]. Each abstract domain is implemented in its own CPA. CPAs in the framework, i.e. value analysis or predicate analysis, can be combined to build an efficient and more precise approach. A configurable algorithm, CEGAR in case of CPALockator, uses CPAs to construct a set of reachable states. In the figure 2 current configuration is presented. The highlighted components are implemented and used only in CPALockator. Lock analysis tracks acquired locks. It helps to compute thread effects that can be applied to a particular thread. Thread analysis determines whether two code blocks may be executed in parallel. Predicate analysis is extended to handle environment actions. It allows constructing a predicate abstraction in a thread-modular case. More information about CPALockator may be found in [1,2].

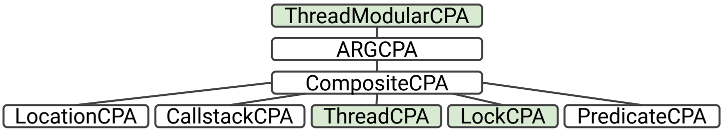

**Fig. 2.** Different CPAs in CPALockator configuration

## 3 Strengths and Weaknesses

First, we need to emphasize that the tool is targeted and used in practice for finding bugs in large industrial software systems, for example, operating system cores. We applied the tool to the Linux kernel and a number of private kernels of real-time OS. The main challenge is scalability there. And results on small but tricky sv-benchmarks look poor, just because of trade-off scalability vs. precision. Our tool is not so precise as other participants, but we show our scalability on a small set of complicated sv-benchmarks. However, it is useful for the community to have such comparison.

The thread-modular approach cannot solve tasks that contain control dependencies in the environment, as we consider all projections independently from each other and thus we lose their order. This is also a problem for witness validation, as the tool provides a path only in a single thread. It is a limitation of the approach, not only the tool itself. In practice we use more user-friendly format to analyze, visualize and evaluate error traces than witness validation [6]. However, the approach allows to simplify thread interaction, and the benefit is considerable for large complicated tasks, which cannot be analyzed with precise model checkers.

As the approach shows benefit for complicated tasks, like in *ldv-linux-3.14-races* directory. CPALockator correctly solves 4 of 7 those benchmarks and for one more obtains an imprecise counterexample. The rest of two tasks may be solved in the other, more faster, CPALockator configuration. The other tools mostly have problems with the benchmarks due to their complexity and size. The explanation of the results is rather evident. Most of the tools try to consider precise interaction between threads, while CPALockator abstracts from it and considers each thread separately. Note, the benchmarks have a strong hint for verifiers: there is only one assert to check while in the real world nobody knows where the bug may be located.

Overall results are not so good because of problems related both to the approach itself and its implementation. The majority of unknowns are related to unsupported atomic operations, like *_atomic_* functions, *compare_and_swap* and so on. Currently, our tool supports only synchronization operations based on locks, as the industrial software mostly contains them. Another problem is related to predicate analysis and interpolation. The current implementation of an interpolation procedure cannot produce interpolants for other threads, which limits the power of predicate analysis. Other problems are also present, but they are not so significant.

Anyway, CPALockator does not produce incorrect **true** verdicts, which confirms the soundness of the approach. All produced **true** verdicts are confirmed by validators, however, its amount is not so numerous, as we skip all tasks with unsupported functions. Thus, the presented approach may be used in combination with more precise techniques.

## 4 Tool Setup and Configuration

We submitted CPALockator[5] built from svn revision `36155` for participation in the category *Concurrency*. The tool requires a Java 11 runtime environment. CPAchecker has to be executed with the following command line:

```
scripts/cpa.sh -svcomp21-lockator -spec reach.prp program.i
```

or via `BenchExec` tool.

## 5 Project and Contributors

The CPAchecker project is mainly developed by an international research group from the Ludwig-Maximilian University of Munich. CPALockator is based on CPAchecker and is developed and supported by researchers from Ivannikov Institute for System Programming of the Russian Academy of Sciences. We thank Dirk Beyer and the CPAchecker team for their work and fruitful discussions.
